# The evolution of a novel approach to building surgical capacity for cervical cancer in Africa

**DOI:** 10.3332/ecancer.2022.1469

**Published:** 2022-11-07

**Authors:** Michael L Hicks, Mulindi Mwanahamuntu, Raleigh Butler, Homer Bloomfield, Alex Mutombo, Mukanya Mpalata Anaclet, Mulumba Kapuka Sylvain, Lameck Chinula, James Kachingwe, Groesbeck P Parham

**Affiliations:** 1 Department of Obstetrics & Gynecology, University of North Carolina at Chapel Hill, Chapel Hill, 101 Manning Dr., Chapel Hill, NC 27514, USA; 2 Department of Obstetrics and Gynaecology, University Teaching Hospital – Women and Newborn Hospital, 10101 Nationalist Way, Lusaka, Zambia; 3 St. Joseph Mercy Oakland, Michigan Cancer Center, 44405 Woodward Ave, Suite 202, Pontiac, MI 48341, USA; 4 Department of Obstetrics and Gynaecology, Princess Margaret Hospital, Nassau, Bahamas; 5 Biamba Marie Mutombo Hospital, No. 9777, Boulevard Lumumba, Commune de Masina, Kinshasa, Democratic Republic of the Congo; 6 Department of Obstetrics and Gynaecology, Kamuzu Central Hospital, Lilongwe, Malawi

**Keywords:** surgical capacity building, novel method of surgical training for early-stage cervical cancer, surgical intensification training programme in Africa, surgical training in LMIC, building surgical capacity in Africa

## Abstract

The human, financial, and infrastructural resources required to effectively treat invasive cancer of the cervix are grossly inadequate in the African region, inclusive of a paucity of surgeons capable of performing life-saving radical pelvic surgery for early-stage disease, and the requisite medical ecosystem (blood banking, anesthesia, laboratory, imaging, diagnostics, etc.) Death without treatment, therefore, is a common sequela of cervical cancer in Africa. As African American gynaecologic oncology sub-specialists working in Africa and its Diaspora, we set out to find a way to alter these circumstances. Herein, we provide an overview of our efforts and how they evolved into a novel method of training that rapidly builds surgical capacity for the treatment of early-stage cervical cancer in resource-constrained environments.

## Introduction

Cervical cancer is the second most common malignancy among women in low-and middle-income countries (LMIC) [1]. Globally, the burden of this reproductive tract cancer is projected to increase to nearly 700,000 cases and 400,000 deaths in 2030, with analogous increases anticipated in future years. Most of the increase will occur in the world’s most impoverished regions, where cancer care is marked by an insufficient cancer workforce, weak diagnostic and imaging capability, limited access to conventional and cutting-edge cancer medicines and meagre health expenditures [2–7].

In sub-Saharan Africa (SSA), cervical cancer ranks as the second leading cause of cancer-related deaths in women, just behind cancer of the breast [1]. The ten countries with the world’s highest cervical cancer incidence rates and deaths are located in SSA *(Global Cancer Observatory: Cancer Today. Lyon, France: International Agency for Research on Cancer. Available from: https://gco.iarc.fr/today, accessed 24 September 2020),* as are the largest global disparities in medical care [1–6]. The number of oncologists in SSA ranges from zero in countries like Lesotho, Benin, Gambia, South Sudan and Sierra Leone to only a few in Malawi, Burkina Faso, Democratic Republic of the Congo (DRC), Rwanda and Togo [4, 5]. The average number of pathologists per capita in Africa is 1/1,000,000, in comparison to 1/20,000 in the USA and UK [6, 7]. Basic infrastructure platforms (electricity, water, clinic space, operating theatres, equipment and supplies, laboratory reagents, etc.) are deficient throughout the region; power and supply shortages are not uncommon and maintenance of equipment (CT scans, MRI machines, linear accelerators, etc.) is unreliable, primarily because of the lack of affordability of deferred maintenance agreements [8, 9]. In addition to the above, a major gap in cancer care in SSA is the lack of surgeons (surgical capacity) capable of performing radical surgical procedures for the treatment of early-stage invasive cervical cancer, as well as other gynaecologic malignancies. This particular therapeutic deficit is part of a problem of greater magnitude, as it is predicted that by 2030 there will be a need for 45 million cancer operations globally; however, less than 5% of LMIC will be able to provide safe, affordable and timely cancer surgery [10, 11]. The above circumstances represent an international public health failure of serious magnitude.

Some of the solutions currently being implemented to close the surgical oncology gap in impoverished nations include the establishment of post-graduate fellowship training programmes within the home countries of trainees; intermittent hands-on training seminars in LMIC by visiting surgical experts from high-income countries (HIC); observerships in HIC where visiting surgeons from LMIC are permitted to observe the clinical and surgical management of patients in HIC, but because of licensure regulations, are unable to engage in hands-on activities; distance training using virtual reality surgical simulations, etc. While all of these approaches have met with some success and deserve to be further developed and expanded, each has its limitations.

Our original premise was to reduce the burden of cervical cancer in women in underdeveloped communities in Africa and its Diaspora. We began our quest in the southern United States through health promotion platforms that emphasised awareness and early detection. Over a 20-year time span, our activities expanded to the Caribbean Islands and later to Africa. During this period, our initial premise evolved into what is now our current implementation approach to women’s cancer control in resource-constrained settings: essential workforce capacity building integrated with simultaneous service delivery of oncologic surgery and clinical infrastructure development. Using a descriptive, explanatory and evaluative approach, we discuss the experiential evolution, adaptation and most recent formulation of our original premise.

### Preliminary activities

#### Health promotion

Cervical cancer and poverty are linked. This is pervasive across Africa and the African Diaspora. In the United States, women living in higher poverty areas experience elevated cervical cancer incidence, morbidity and mortality rates [12–14]. The majority of these geographic settings are located in the southern region of the nation, where the labor of enslaved Africans originally served as the primary source of wealth generation and fueled the economic engine of the nation. It is these same areas that are now highly characterised by a lack of access to care and outreach programmes for women of all racial and ethnic groups, particularly those of African descent [12]. In previous publications, we documented the gross disparities in medical care for African American women diagnosed with gynaecologic malignancies [14–17].

In our approach to affect change, one of the authors (MH) worked in a rural community in the Alabama ‘Black Belt’ in the mid-1980s, performing Pap smear-based cervical cancer screening in the basements of churches in the African American community, as African Americans had very limited access to private and public healthcare because of persistent ‘Jim Crow’ attitudes and policies of exclusion based on race (Table 1). In the early 1990s, two of the authors (GP and MH) began a series of educational seminars labelled ‘Town Hall Talks’ in African American communities in the rural southern states of Alabama, Arkansas, Mississippi and Louisiana, all of which have excessively high rates of lifestyle-associated cancers [12] (Table 1). The goal of the educational activities was to personally convey information and make recommendations about lifestyle changes that could decrease cancer risks, as well as provide guidance on cancer screenings and early detection. The complexity of practicing medicine and acquiring hospital privileges across state lines were impediments to personally providing clinical care in these states. We therefore relied on education alone as the sole imperative (Table 2).

#### Live demonstration projects

Widespread knowledge of our health promotion activities in the southern U.S. resulted in an invitation to visit the Caribbean Islands, a global setting also characterised by high rates of cervical cancer. There we implemented cervical cancer prevention educational seminars using visual inspection with acetic acid (VIA) for cervical cancer screening and loop electrical excision procedure (LEEP) for the treatment of precancerous lesions. Our audience primarily consisted of healthcare providers in the Bahamas, Barbados, St. Lucia and Jamaica from 1998 to 2001 (Table 1). These activities led to the establishment of valuable personal relationships and a collaboration with the first formally trained gynaecologic oncologist in the Bahamas (RB). At his request, and with the intent of developing sustainable gynaecologic oncology capacity, we began implementing quarterly on-site visits to the Bahamas, during which times we performed live surgical demonstrations on complex gynaecologic oncology cases, previously selected by our local host (Table 1). Through these hands-on clinical educational activities, we successfully enhanced the surgical oncology skills of our Bahamian colleague. With the later addition of another native Bahamian who had been formally trained in gynaecologic oncology in Canada, gynaecologic oncology subspeciality services were expanded and became sustainable in the Bahamas, lessening their dependence on our intermittent visits. Currently, state-of-the-art gynaecologic oncology services are offered in the Bahamas with three formally trained gynaecologic oncologists, with a fully integrated gynaecologic oncology fellowship training programme through the auspices of the International Gynecologic Cancer Society (IGCS) directed by (RB). We (GP, MH) actively participate in their monthly Extension for Community Healthcare Outcomes (ECHO) Multidisciplinary Tumor Board (Table 2).

#### Africa

With the experiences and lessons learned in the Bahamas, we expanded our training activities to South Africa at the Natalspruit Hospital (now Thelle Mogoerane Hospital), University of Natal Durban (now University of KwaZulu-Natal) and the Medical University of South Africa - MEDUNSA (now Sefako Makgatho Health Sciences University - SMU), where we continued to perform live surgical demonstrations on complicated gynaecologic oncology cases from 2001 to 2002 (Table 1). The premise of our activities in South Africa was the same as in the Bahamas; that through intermittent (1 week) visits during which time we performed surgery with our local colleagues, we could train mid-level general gynaecologists how to perform some of the critical aspects of complicated gynaecologic cancer surgery, resulting in the establishment of local gynaecologic oncology surgical capacity. What we observed during this time period were the following circumstances that impeded the realisation of our premise: 1) Some of the general gynaecologists we attempted to train were consumed with obstetrical duties and demands of private practice, and thus were unable to bring the degree of focus to the training seminars that was required for effective skills transfer; 2) Unlike in the Bahamas, none of the general gynaecologists we worked with during this period in South Africa had extensive experience in complex gynaecologic surgical concepts or formal training in gynaecologic oncology; 3) There were a variety of different type of cancer cases ( ovarian, vulva, cervix and endometrial) each requiring a different set of surgical skills, which did not permit sufficient surgical repetition of a single set of surgical procedures for the treatment of one particular cancer type for the trainees; and 4) Our intermittent visits were spaced out too far apart, thereby diluting the intensity of the surgical training. The only exception to 1–3 above was at the Medical University of South Africa – MEDUNSA (now Sefako Makgatho Health Sciences University – SMU), where there was a gynaecologic oncologist, but the infrequency of our visits was still a major impediment.

Although we travelled to these various facilities with the intention of building or enhancing surgical capacity, the above circumstances jettisoned our presupposition for building gynaecologic oncologic surgical capacity using this particular methodology (Table 2).

In 2003, we were invited to Lusaka, Zambia where we collaborated with local gynaecologic colleagues at the University Teaching Hospital (UTH) to host educational seminars on cervical cancer screening; assisted with the establishment of the first colposcopy clinic; and again performed surgical demonstrations (Table 1). In Zambia, we quickly established a relationship with a general gynaecologist (MM) who had advanced pelvic surgical training, and a very keen interest in learning how to perform radical gynaecologic cancer surgery. Additionally, he and other clinicians at UTH were not preoccupied with other clinical distractions (e.g. private practice) that prevented focused learning, as had been the case with our colleagues in South Africa. Another important variable in Zambia was our ability to personally connect with the practitioners and ancillary staff. This led to trust, mutual respect and understanding; resulting in a solid foundation for future professional collaborations. During each visit to Zambia, the local trainee performed multiple cases (5–6) of radical hysterectomy over a 1-week interval, with us acting as the first assistant, which facilitated a more rapid transfer of knowledge/skills. The rapid transfer of surgical skills via the performance of multiple cases of one specific surgical procedure (radical hysterectomy), over a short period of time, we labeled as ‘focused surgical intensification’. This led to the postulation that such an approach could in fact represent a method for rapid surgical capacity building in resource-constrained environments. One of the authors (GP) established full-time residency in Zambia in 2005 and joined the hospital staff, allowing the Zambian relationship to be sustained, for now close to 20 years. The country presently hosts a full gynaecologic oncology service infrastructure with a referral centre and training fellowship, initially through the auspices of IGCS, but now an integral part of the University of Zambia (Masters of Medicine) MMed postgraduate programme. While (MM) serves as the local mentor for the programme, (GP) and (MH) are international mentors; (GP) is an Honorary Consultant at UTH and (ML) is a visiting Professor. Because of the above relationship by (GP) along with frequent visits by (MH), Zambia served as the catalyst of our shift in approach to finding a better and faster way of building gynaecologic oncology surgical capacity in Africa.

The approach of ‘focused surgical intensification’ that evolved over time consists of a matrix of pre-site review of multimedia surgical material by local general gynaecology trainees, onsite didactic and hands-on training led by visiting senior gynaecologic oncologists, post-visit Internet follow-up communication and continued e-learning. The specifics of this constructive experiential derived novel training programme are described below.

## Methods

### Teaching model

The core of the model is that experienced gynaecologic oncologists train mid-level general gynaecologists to perform a single surgical procedure (focused) – radical abdominal hysterectomy, bilateral pelvic lymphadenectomy – using high volume repetition (several cases/day) of the procedure over a short time interval (surgical intensification). The curriculum is competency-based and consists of the following: (1) Self-directed learning of recommended material (prior to, during and post visit); (2) preoperative clinical evaluation of all patients scheduled for surgery; (3) multimedia evaluation of the surgical procedure and anatomy prior to each surgical case; (4) intra-operative verbal and anatomical mentoring, during which time trainees are repeatedly shown and instructed how to perform the most critical subcomponents of the surgical procedure in a sequential fashion by the gynaecologic oncology trainers, and the rationale behind each step; (5) immediate post-operative assessment of the trainee’s ability to perform each critical component of the operation; (6) trainee’s reflection and review of the surgical procedure, including identification of potential intra-operative surgical complications (injury to bowel, urinary bladder, ureters and major regional blood vessels) with explanations of viable corrective surgical measures; (7) postoperative care and follow-up recommendations specific to each respective patient; (8) preoperative, intra-operative and post-operative assessment of the trainee by gynaecologic oncology trainers to determine the trainee’s level of proficiency in performing the radical hysterectomy with pelvic lymphadenectomy independently. In order to promote proper governance of a complex surgical service, we stressed the importance of ethical behaviour/decorum in the operating room, strict attentiveness to details and the elimination of any potential distractions. The importance of maintaining a balanced mental temperament and respectful demeanour was emphasised.

### Training sites

The geographic sites for the above training model of ‘focused surgical intensification’ were as follows: UTH in Lusaka, Zambia (with MM); Kamuzu Central Hospital in Lilongwe, Malawi (with LC and JK), Biamba Marie Mutombo Hospital, in Kinshasa, DRC (with AM, MMA, MKS), respectively. The hospitals in Zambia and Malawi were public sector facilities while the hospital in the DRC is a private sector healthcare facility. In each, a cervical cancer screening programme was established, from which most of the early-stage cervical cancer cases originated [17–19] (Tables 3 and 4).

### Personnel

All surgical trainees were board-certified obstetrician/ gynaecologists who expressed an avid and fervent interest in learning how to perform a radical abdominal hysterectomy and pelvic lymphadenectomy. They were selected by their respective institutions to be part of the focused surgical intensification programme, with the goal of eventually developing a clinical service for the surgical treatment of early-stage cervical cancer within their home institutions. The cervical cancer master trainers were two U.S. board-certified gynaecologic oncologists (MH, GP) with over 50 years of surgical experience between them, much of which has been spent training and practicing medicine and teaching in U.S. locations with high cervical cancer burdens (Detroit, Michigan; South Central Los Angeles, California; Little Rock, Arkansas; Birmingham, Alabama). Over the past 20 plus years, they have worked together in SSA and the Caribbean islands leading cervical cancer screening and surgical oncology demonstration seminars (Table 1). Both were trained during a period when open laparotomy was the standard approach for the treatment of early-stage cervical cancer.

## Results

During the period of primarily performing surgical demonstrations, we were able to successfully assist general gynaecologists with very complex gynaecologic oncology surgical procedures. Table 1 shows the variety of procedures that were performed in each location.

From 2012 to 2019, we implemented the focused surgical intensification training curriculum, specifically geared to teach the radical hysterectomy, pelvic lymphadenectomy. Table 3 shows the site locations, type of physicians trained, specific objectives, adjunct benefits, outcomes and time period of the programmes. Table 4 shows there were a total of 72 radical hysterectomies, pelvic lymphadenectomies performed during the training period, in three different countries in Africa. The number of cases needed for the trainee to develop the ability to independently perform the radical hysterectomy ranged from 6 to 8 cases. Major complications ranged from 2 to 4 occurrences, for a total of 8 (11%) out of the 72 cases. To date, 490 radical hysterectomies have been collectively performed, with the Zambian programme (original site), having performed the majority of these cases.

## Discussion

As African American gynaecologic oncologists, we set out to find ways to abate the burden of cancer in women from Africa and its Diaspora. Using educational seminars (Town Hall Talks) as our initial approach, we began our activities in the African Diaspora in the United States, in former slave states, where the overall rates of cancer deaths are higher [12]. These educational seminars were well attended and temporarily had success in conveying information about risk behaviours. We attributed much of the programme’s success to the fact that we were African American physicians with deep roots in the target communities – the impoverished African Diaspora in America – and to our communication skills. The Town Hall Talks were short-lived due to lack of funding. Additionally, we learned that health promotion activities alone had limited impact in reducing the burden of cervical cancer.

The next phase of our activities marked the beginning of intermittent on-site training visits, sometimes referred to as ‘parachuting’. During this period, we began performing surgical demonstrations in the Bahamas and South Africa. In both countries, we were able to make an immediate impact on patients by performing complex oncologic surgical procedures, that otherwise could and would not have been performed (Table 1). Although rewarding, beneficial and unequivocally necessary, we realised in South Africa that this method was not sustainable and would not lead to the development of local gynaecologic oncology surgical capacity (Table 2). This resulted in an evolution towards experientially developing a new training paradigm that could be customised for surgical capacity building in resource-constrained settings.

After more than a decade of travel through Africa, performing surgical demonstrations with local gynaecologists, making adjustments to overcome programmatic flaws and buttressing local infrastructure and health system weaknesses, we arrived at the formulation, development and implementation of ‘competency-based focused surgical intensification’, a programmatic approach specifically designed for the rapid transfer of surgical skills for the radical abdominal hysterectomy and pelvic lymphadenectomy, as described in the methods section. From 2012 to 2014, we successfully used this approach to train gynaecologists in Zambia (Tables 3 and 4). The transfer of knowledge and skills necessary to perform this particular surgical procedure was rapid, taking only 6–8 cases over a period of 5 days, to achieve proficiency (Table 4). These results led us to utilise the same novel training concept in Malawi and most recently in the DRC (Tables 3 and 4) [17, 18]. Table 4 shows how quickly the level of competency was achieved along with the number of cases performed during the training programme at the respective sites. Additionally, it shows the impact based on the overall number of cases performed to date at the collective sites.

All three of these countries have some of the highest cervical cancer incidence rates in the world [1]. Using a nontraditional approach to surgical training that takes advantage of high volumes of disease, we significantly accelerated the usual time required for a trainee to master the skills necessary to perform a potential curative surgical procedure. The result was the production of local surgeons capable of successfully performing a single radical oncologic surgical intervention, and the immediate establishment of new surgical oncology services (capacity) that were previously nonexistent, a novel concept used to build gynaecologic oncology surgical capacity in resource-constrained environments.

To the authors’ knowledge, there are eight official training programmes for gynaecologic oncology in Africa (Ethiopia, Kenya, Ghana, Uganda, Mozambique, Zambia, South Africa and Rwanda) and one too soon start in Nigeria [20]. Each programme has 2–3 fellows and the time of training ranges from 2 to 3 years in length. Although we acknowledge and support this form of training, and three of the authors (GP, MH and RB) participate in this training format, because of significant deficiencies in the surgical ecosystems, much remains to be achieved when exclusively using this form of building gynaecologic oncology surgical capacity in Africa, suggesting the need for other innovative strategies that are complementary.

The requirements for gynaecologic oncology fellows in the U.S. for cervical cancer treatment is set at the minimum number of ten cases per fellow (inclusive of minimally invasive and open surgical approaches, and radiation treatment with brachytherapy) over the course of their 3-year fellowship training (Accreditation Council for Graduate Medical Education, 2018). Historically over the past 10–15 years, many of the U.S. gynaecologic oncology fellows in training perform only 5–10 radical hysterectomies during their entire 2–3 years of clinical fellowship training; until recently, the majority of these were done robotically and not in a repetitive fashion. Therefore, their experience with performing the abdominal radical hysterectomy is even more limited. Lastly, unlike in most U.S. facilities, in Africa, there is no or limited radiation available for the treatment of cervical cancer, so radical hysterectomies are often performed on cervical lesions that are larger than 4 cm with no parametrial involvement. Our trainees reached the level of competency to independently perform the operation at the same minimal levels (6–8 cases) of what is acceptable for U.S. gynaecologic oncology fellowship training programmes. However, our trainees performed far more cases on an individual basis, and in a much shorter period of time, which added to their confidence and proficiency.

The focused surgical intensification approach facilitated the creation of immediate in-country gynaecologic oncology surgical capacity for the first time in three countries in SSA. Additionally, in the DRC, we were able to develop a women’s cancer centre through simultaneous training and service delivery of oncologic surgery, development of clinical infrastructure and chemotherapy infusion.

Our trainees have collectively gone on to perform hundreds of radical hysterectomies and are now training other general gynaecologists, thereby improving in-country capacity for this operation resulting in lives saved that were once lost (Table 4).

Despite its successes, our approach to training has its own challenges, as demonstrated in Table 5. However, despite these difficulties, we suggest this model of training should be considered and supported by countries that are in need of immediate gynaecologic oncology surgical capacity, and used as a bridge in the establishment of full-fledged gynaecologic oncology fellowships, and as an adjunct by large organisations that are charged to eradicate cervical cancer in LMIC.

## Conclusion

We have presented our experience of building gynaecologic oncology surgical capacity in Africa, through a novel concept that is customised for resource-constrained regions of the world with heavy cervical cancer burdens. Through our learned experience and a labour intense process, we would like the reader to know that there are many intangible items that will be encountered, and unimaginable challenges to overcome in order to achieve these outcomes. For them to be conquered requires love, compassion, empathy and the seraphic gift of constant improvisation with no judgement or anger; which can only be experienced through the mentors’ unwavering commitment to service and the recipients’ genuine trust in their senior trainers, which are ethereal entities that are impossible to elucidate, chronicle and tabulate in a customary corporeal written manuscript.

## Conflicts of interest

None.

## Funding

None.

## Figures and Tables

**Table 1.** Educational seminars and surgical demonstration programmes.

Country	Educational seminars	Surgical demonstrations	Time period	Purpose/objective	Type of cases
United States	Yes	No	1985–2001	Pap smear-based cervical cancer screening in the basements of churches in the African American community (Alabama Black Belt) Community education via educational seminars (Arkansas, Alabama, Mississippi, Louisiana)	None
Caribbean Islands	Yes	Yes	1998–2001	Educational seminars (Bahamas, Barbados, St. Lucia and Jamaica) Improving gynaecologic oncology surgical skills (Bahamas)	Ovarian cancer debulkings, radical hysterectomy, pelvic lymphadenectomy, radical vulvectomy and inguinal-femoral lymphadenectomy, bowel resections; ileal conduits and ureteral reimplantations (Bahamas only)
South Africa	Yes	Yes	2001–2002	Improving gynaecologic oncology surgical skills	Ovarian cancer debulkings, radical hysterectomy, pelvic lymphadenectomy, radical vulvectomy and inguinal-femoral lymphadenectomy, bowel resections; ileal conduits and ureteral reimplantations
Zambia	Yes	Yes	2001–2012	Educational seminars Improving gynaecologic oncology surgical skills	Ovarian cancer debulkings, radical hysterectomy, pelvic lymphadenectomy, radical vulvectomy and inguinal-femoral lymphadenectomy, bowel resections; ileal conduits and ureteral reimplantations

**Table 2.** First phase of premise to build surgical capacity in the African Diaspora and Africa.

Location	Hypothesis for capacity building supported	Hypothesis for capacity building rejected	Impediments	Benefits
American African Diaspora	No	Yes	1. Illegal to operate across state lines 2. Unable to obtain immediate hospital privileges across state lines 3. Unable to obtain immediate liability insurance protection or protection waiver across state lines	Community education on cancer screening and lifestyle changes to potentially reduce the risk of cancer
Bahamas	Yes	No	None	1. Personal desire to develop a sustainable oncological surgical service 2. Proper echo-system (anaesthesia, blood bank, laboratory services, pathology, governmental support, theatre space and time, new trainees) 3. Formally trained gynaecologic oncologist in country 4. No competing clinical impediments 5. Full service gynaecologic oncology services 6. Gynaecologic oncology fellowship
South Africa	No	Yes	1. Clinicians with other competing clinical responsibilities (obstetrical and private practice demands) 2. Because of #1 there was limited time to dedicate to learning the complex surgical procedures 3. Intermittent visit were too infrequent 4. Lack of focus on one complex surgical procedure to master	Able to perform much needed surgery in this region, that benefited each patient personally

**Table 3.** Description of programmes for gynaecologic oncology capacity building.

Country	Type of physician	Specific objective of training	Adjunct surgery	Outcome of the programme	Time period
Zambia	General gynaecologist with informal training in gynaecologic oncology	Rapidly teach the trainee how to perform a radical hysterectomy, pelvic lymphadenectomy for early-stage cervical cancer	Ovarian cancer debulkings, radical hysterectomy, pelvic lymphadenectomy, radical vulvectomy and inguinal-femoral lymphadenectomy, bowel sections; ileal conduits and ureteral reimplantations	1. Rapid transfer of surgical skills for radical hysterectomy, pelvic lymphadenectomy for the treatment of early-stage cervical cancer 2. Enhanced skills in doing other complicated gynaecologic oncology surgery and complex pelvic surgery (i.e. Caesarean hysterectomy for postpartum haemorrhage) 3. The development of an international gynaecologic oncology fellowship programme 4. Sustainability of a gynaecologic oncology service 5. The ability to train new gynaecologic oncologist 6. Transfer of knowledge of a life-saving surgical procedure	2012–2019
Malawi	General gynaecologist	Rapidly teach the trainee how to perform a radical hysterectomy, pelvic lymphadenectomy for early-stage cervical cancer	None, programme was specifically geared to teach the radical hysterectomy and pelvic lymphadenectomy	1. Rapid transfer of surgical skills for radical hysterectomy, pelvic lymphadenectomy for the treatment of early-stage cervical cancer 2. Interest in starting an international gynaecologic oncology fellowship programme 3. The ability to train other general gynaecologist to perform this specific operation 4. Transfer of knowledge of a life-saving surgical procedure	2015–2017
DRC	General gynaecologist	Rapidly teach the trainee how to perform a radical hysterectomy, pelvic lymphadenectomy for early-stage cervical cancer	Ovarian cancer debulkings, radical hysterectomy, pelvic lymphadenectomy, radical vulvectomy and inguinal-femoral lymphadenectomy, bowel resections; vesico-vaginal fistula repair and ureteral reimplantations	1. Rapid transfer of surgical skills for radical hysterectomy, pelvic lymphadenectomy for the treatment of early-stage cervical cancer 2. Enhanced skills in doing other complicated gynaecologic oncology surgery and complex pelvic surgery (i.e. Caesarean hysterectomy for postpartum haemorrhage) 3. Sustainability of a gynaecologic oncology service 4. The ability to train other general gynaecologist to perform this specific operation 5. Transfer of knowledge of a life-saving surgical procedure 6. The establishment of a woman’s cancer centre inclusive of chemotherapy infusion	2017–2019

**Table 4.** Surgical numbers and complications.

Country	Number of cases of radical hysterectomies, pelvic lymphadenectomies during the training period	Cases needed to gain minimal proficiency (point at which the trainee is able to perform the surgery independently)	Major complications#(%) during training period	Cases through 2022
Zambia 2012–2014	25	6	2 (8)	360
Malawi 2015–2017	28	7	2 (7)	75
Democratic Republic of Congo 2017–2019	19	8	4 (21)	55
Total	72	Average cases for proficiency 7	Complication rate 11%	490

**Table 5.** Challenges and solutions.

Challenges	Solutions
Multiple site visit	Travel also linked with distant electronic learning
Blood banking	Mobilising family members for donation prior to surgery
Anaesthesia availability	Planning and communication with anaesthesia preoperatively and the use of nurse anaesthetist
Limited antibiotics	No viable solution secondary to the financial circumstances of the patients and limited local supply of antibiotics
Surgical theatre time	Preplanning for utilisation
Surgical instrumentation	Purchased new surgical instruments
Extreme poverty affecting travel	No viable solution secondary to the financial circumstances of the patients
Language and cultural barriers	Begin to learn the local language and respect cultural norms
